# Rendezvous endovascular common carotid artery stenting (RECCAS) technique for symptomatic steno-occlusive disease

**DOI:** 10.1186/s42155-020-00194-3

**Published:** 2021-01-18

**Authors:** M. T. Wang, M. Schembri, H. K. Kok, J. Maingard, M. Foo, A. Lamanna, M. Brooks, H. Asadi

**Affiliations:** 1 Interventional Radiology and Neurointerventional Services, Department of Radiology, Austin Health, Melbourne, Australia; 2 Interventional Radiology Service, Department of Radiology, Northern Health, Melbourne, Australia; 3 School of Medicine, Deakin University, Melbourne, Australia; 4 Interventional Radiology and Neurointerventional Services, Department of Radiology, Monash Health, Melbourne, Australia

**Keywords:** Common carotid artery, Stenting, Steno-occlusive, Technique

## Abstract

This report describes a patient who presented with acute but transient right arm weakness and altered sensation secondary to severe stenosis of the left common carotid artery (CCA) origin. Endovascular stenting of the stenosed origin was achieved utilising a novel rendezvous technique through combined retrograde common carotid artery and anterograde transfemoral approaches. This technique has numerous potential advantages over traditional transfemoral endovascular and open retrograde common carotid artery approaches. It allows increased procedural control and success in traversing the stenosis and provides a smooth transition for the stent delivery catheter. An open cutdown procedure or open surgical technique is not required. Our patient recovered well from the procedure with no complications within the three-month follow up period.

## Background

Extracranial carotid artery steno-occlusive disease is a major cause of recurrent ischaemic stroke, accounting for approximately 20% of all strokes.(Veith et al., 2001) The incidence of significant stenosis or occlusion affecting the origins of the aortic arch branch vessels is 0.5–6.4%.(van de Weijer et al., 2015) The vast majority of current literature is focused on the management of internal carotid artery disease, with a relative paucity regarding the management of common carotid artery steno-occlusive disease. The treatment options for internal carotid artery steno-occlusive disease include carotid artery stenting and carotid artery endarterectomy.(Liapis et al., 2019) The evidence regarding the management options for common carotid artery (CCA) steno-occlusive disease is more controversial,(Klonaris et al., 2013) however the European Society for Vascular Surgery 2017 guidelines now recommend open retrograde stenting for symptomatic isolated common carotid stenoses (Level C).(Naylor et al., 2018)

A retrograde CCA approach allows for easier passage across the site of stenosis into the aortic arch compared with antegrade techniques, most commonly via the common femoral artery.(Makaloski et al., 2017; Paukovits et al., 2008) The retrograde CCA technique requires extensive dilatation of the common carotid artery which typically warrants an open surgical exposure(Samaniego et al., 2015) as well as the passage of the distal end of the stent through a tightly stenosed common carotid artery origin. Conversely, the transfemoral approach may be unsuccessful in traversing the CCA lesion, particularly if it is flush with the aortic arch.(van de Weijer et al., 2015)

We present a case describing a novel antegrade-retrograde rendezvous technique for stenting the left CCA origin that overcomes the aforementioned difficulties utilising a combined retrograde CCA and antegrade CFA approach in a patient with symptomatic severe left common carotid artery origin stenosis.

## Main text

**Fig. 1:**
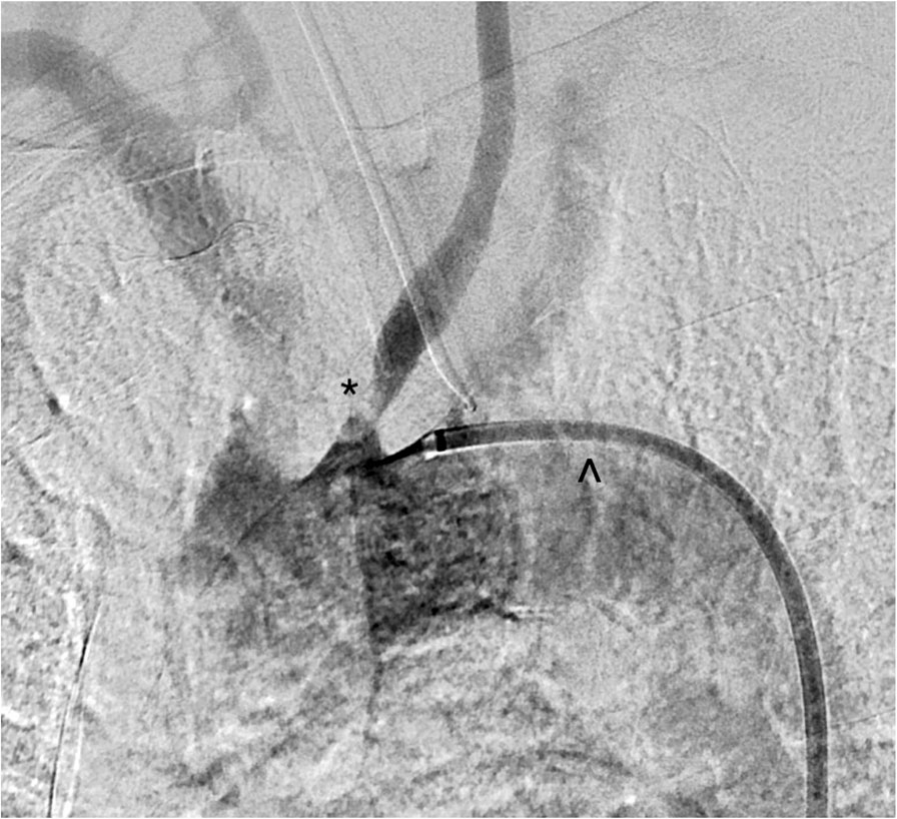
Catheter angiogram demonstrating the stenosed left common carotid artery origin (*). The Neuron Max catheter (^) tip is situated near the ostium of the left common carotid artery

A 76-year-old male presented with transient acute onset right arm weakness and altered sensation. CT extracranial angiography demonstrated high grade stenosis (> 90%) of the left common carotid artery origin (Fig. 1) without significant left internal carotid artery or bifurcation disease. The patient was prescribed a loading dose of aspirin 300 mg, followed by continuation of dual antiplatelet therapy consisting of aspirin 100 mg and clopidogrel 75 mg daily which was commenced following a right MCA stroke several years prior. Given the proximal location of the stenosis at the CCA origin, the lesion was stented by the interventional neuroradiology team utilising a rendezvous technique to traverse the stenotic origin and provide robust guidewire support for stent delivery with the primary aim of preventing further arterial embolic events.

The procedure was performed with the patient under general anaesthesia with IV heparin 7000 IU (80 IU/kg) administered at the start of the procedure. Right CFA access was obtained with placement of an 8 French introducer sheath and a 6 French Neuron Max catheter (Penumbra Inc., Alameda, California) was advanced to the aortic arch.

**Fig. 2:**
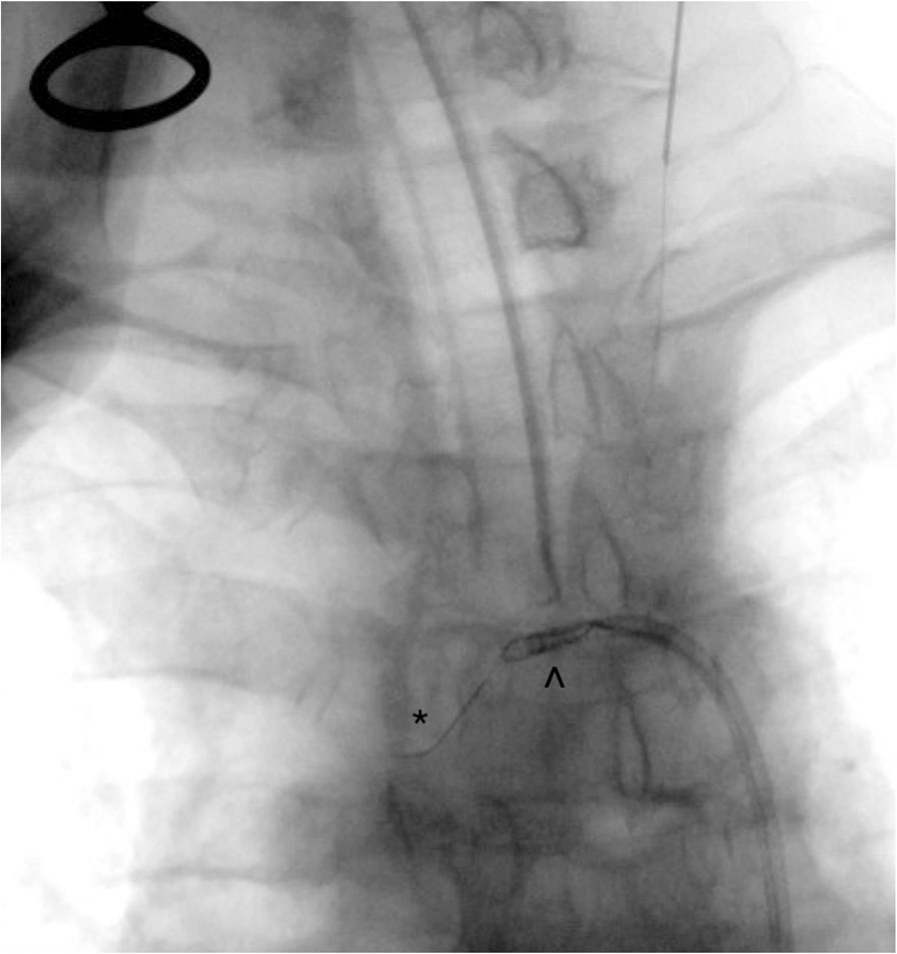
The glidewire (*) inserted through the CCA puncture site is snared (^) into the Neuron Max. This glidewire is then retracted through the femoral puncture site, allowing control from both ends

An ultrasound guided retrograde left CCA puncture was performed with a 21G, 7 cm micropuncture needle (Micropuncture Access Set, Cook Medical, Indiana, USA). The needle was inserted into the superior third of the left common carotid artery, with a 300 cm length 0.014″ guidewire (Glidewire Advantage, Terumo, Tokyo, Japan) advanced through the micropuncture needle. No sheath was utilised. The 0.014″ guidewire was then used to cross the stenosed left CCA origin into the aortic arch. The guidewire was snared into the Neuron Max catheter in the aortic arch using a 2 mm × 175 cm AndraSnare Micro ASM-2 (Andramed, Reutlingen, Germany) (Fig. 2) and its tip retrieved outside the patient achieving through and through carotid-femoral access. This enabled control and access to both ends of the guidewire across the CCA lesion, whilst also enabling gentle tension to be applied to the wire from both the CCA and CFA access sites for maximum support.

**Fig. 3:**
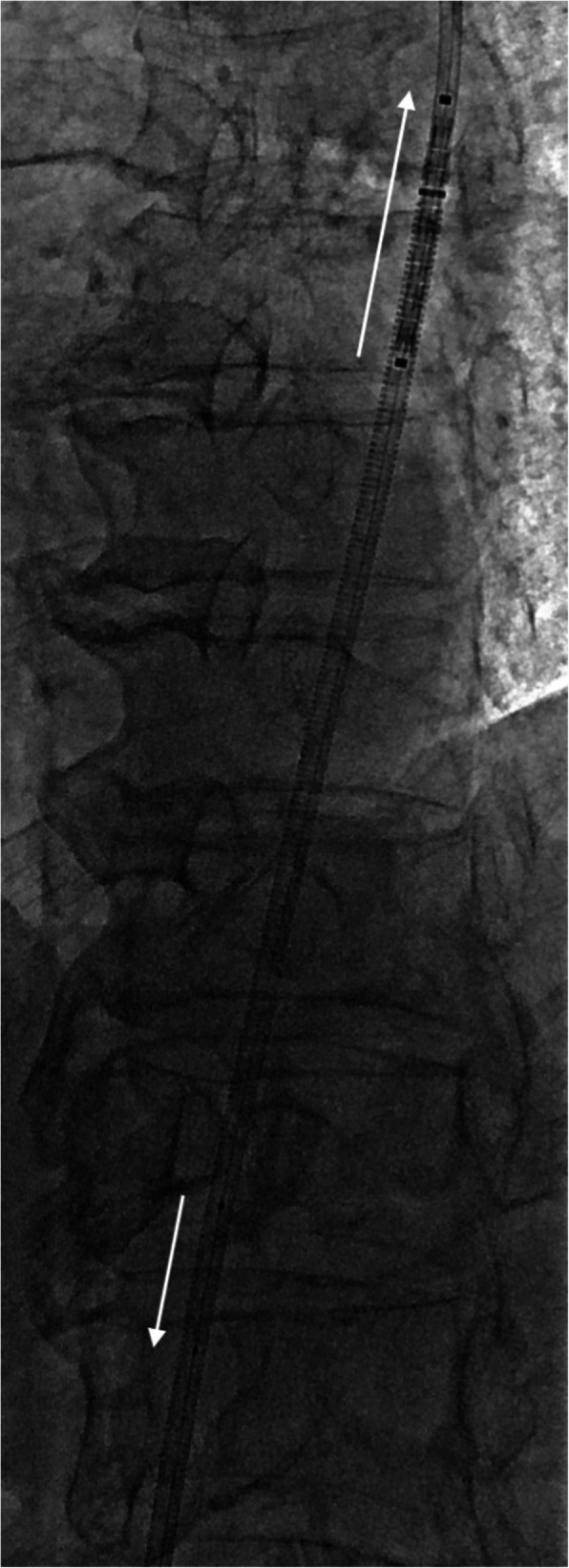
The stent (↑) is advanced anterogradely through the Neuron max over an uninflated Coyote balloon catheter inserted retrogradely (↓) through the CCA puncture site. The uninflated balloon catheter largely occludes the stent lumen

**Fig. 4:**
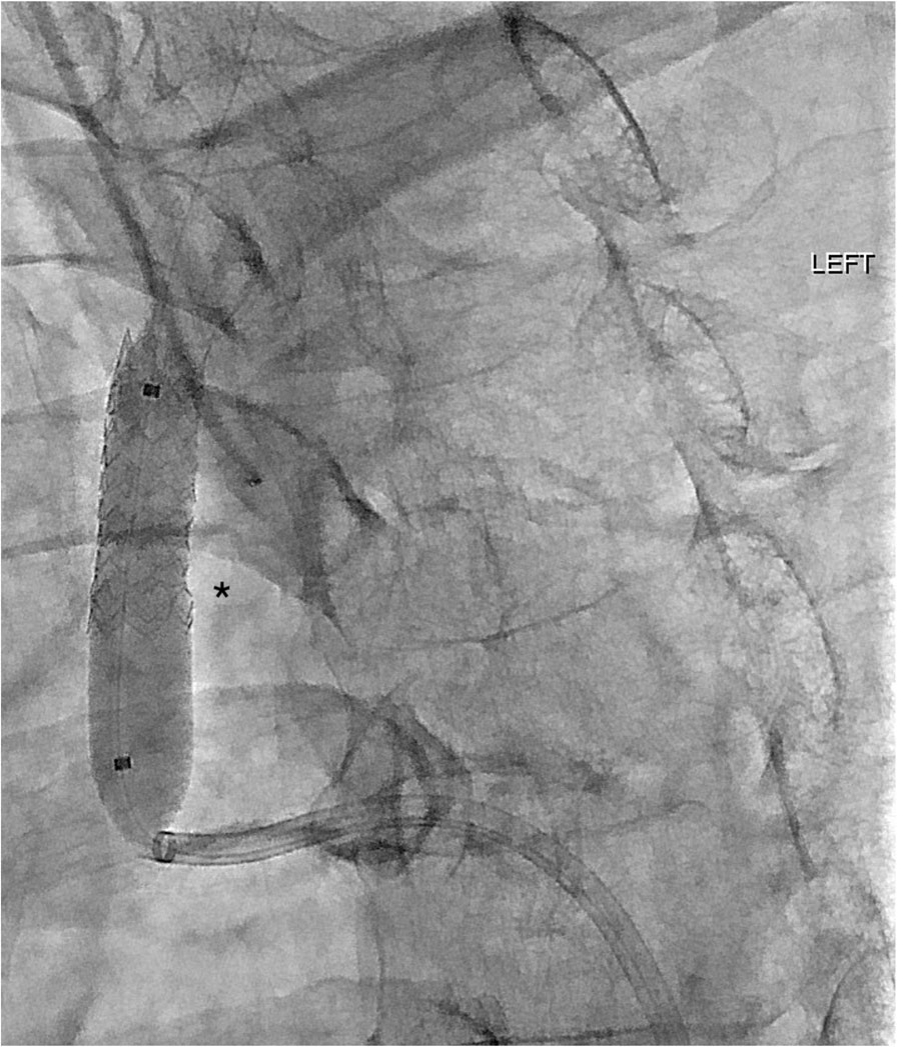
Balloon remodelling of the CCA stent (*). The proximal aspect of the stent is slightly flared at the CCA ostium

An 8 mm × 27 mm BeGraft balloon-mounted expandable laser cut covered stent on a 0.035″ delivery platform (Bentley, Hechingen, Germany) was advanced antegradely from the femoral access site. Following this, a non-inflated 3 mm × 20 mm Coyote balloon catheter (Boston Scientific, Marlborough, Massachusetts) was navigated retrogradely over-the-wire through the left CCA access site and advanced into the stent delivery catheter, occluding its lumen and creating a smooth transition aimed at facilitating tracking of the stent when navigating across the CCA origin stenosis (Fig. 3). The BeGraft stent was easily passed across the stenosis whilst simultaneously retracting the Coyote balloon catheter and applying gentle traction to both ends of the wire for support. After optimal positioning, the stent was deployed and balloon remodelled with the inferior end flared outwards at the CCA ostium. (Fig. 4). Conclusion angiogram demonstrated a widely patent left CCA origin with no embolic complication on ipsilateral cerebral angiography.

**Fig. 5:**
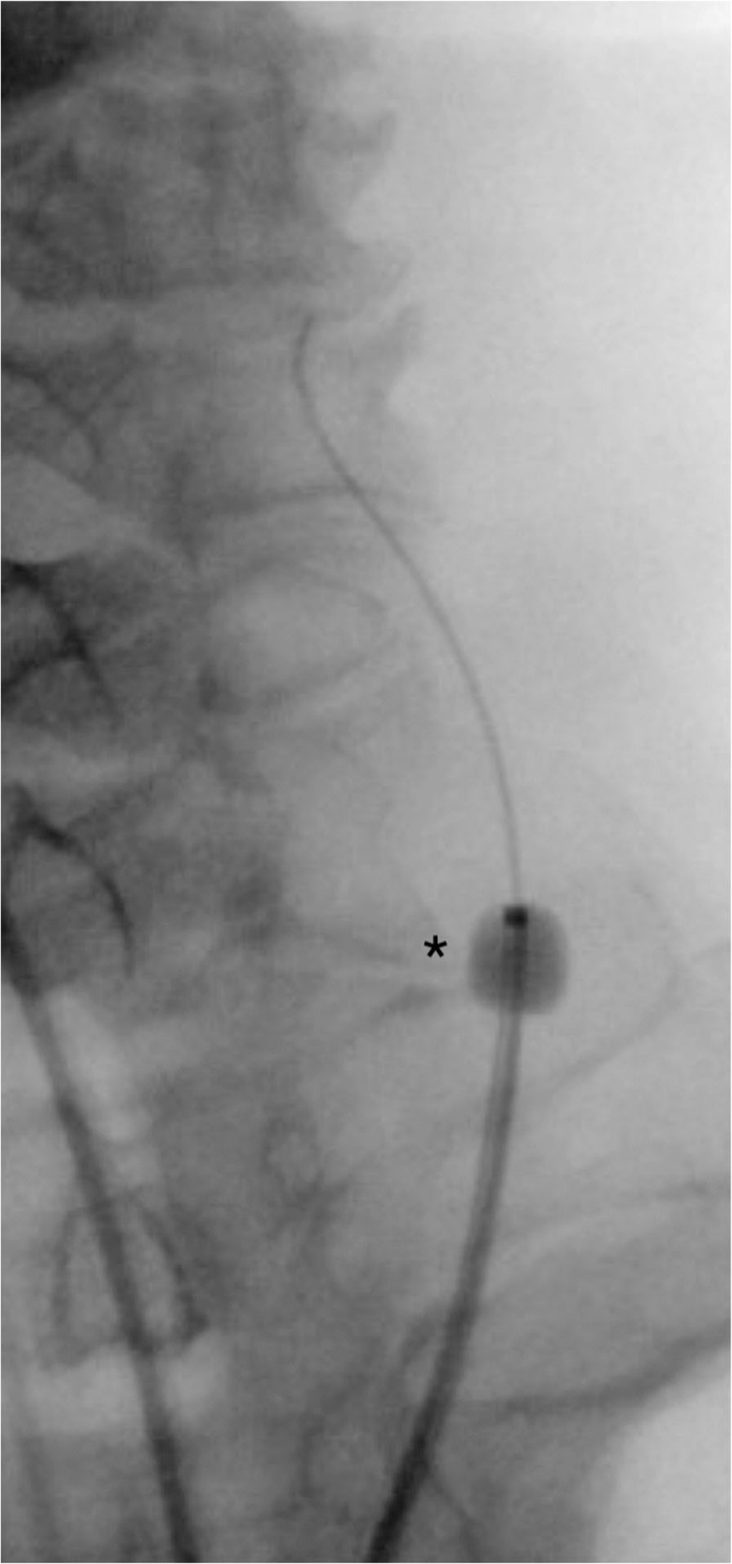
Balloon occlusion of the CCA puncture site with a Corail occlusion balloon catheter (*)

Haemostasis of the CCA puncture site was achieved with a combination of gentle external ultrasound-guided compression and a 6-French 11 ml Corail occlusion balloon catheter (Balt, Montmorency, France) inflated for 3 min (Fig. 5). The Corail occlusion balloon catheter was advanced anterogradely through the CFA, and inflated at the time of removal of the microwire. After removal of the wire, catheter and sheath, femoral haemostasis was secured with an 8-French Angio-Seal (Terumo, Tokyo, Japan) vascular closure device.

The patient recovered well from the procedure with continuation of dual antiplatelet therapy and was discharged 2 days post-procedure. No new symptoms or complications were identified within the initial three-month follow up period.

Symptomatic CCA disease has a significant morbidity primarily due to its association with recurrent ischaemic strokes.(Veith et al., 2001) The trend towards stenting symptomatic CCA steno-occlusive disease is supported by recent guidelines, however the quality of evidence remains mixed,(Naylor et al., 2018) and the optimal technique for CCA stenting unclear. CCA stents should be positioned with a slight protrusion into the aorta to ensure acceptable covering of the ostium and is particularly important for true ostial lesions.(Maleux & Nevelsteen, 2002)

We hypothesize several advantages over traditional techniques in patients with severe stenoses, particularly at the CCA origin, which may be technically challenging via a transfemoral approach.

Avoiding tortuosity of access vessels from the common femoral to the aortic arch allows more direct control of the microwire as it crosses the common carotid stenosis. Having control of tension in both ends of the wire allows more accurate positioning of the stent.

Passing the balloon expandable stent over a smaller, uninflated balloon catheter provides a smooth transition between 0.014″ wire and the 0.035″ in stent delivery system, enabling the lumen of the balloon expandable stent delivery catheter to be mostly occluded during device navigation and potentially reducing the risk of atherosclerotic plaque disruption as the stent is advanced antegradely across the stenosis.

Lastly, by reducing the size of access required in the CCA, the rendezvous technique allows for safe and effective haemostasis to be achieved with a combination of manual compression and temporary balloon occlusion at the puncture site, eliminating the need for a vascular closure device or an open surgical ‘cut down’ approach to access the common carotid .

This technique did not employ predilatation or use of a cerebral protection device. Current guidelines support predilatation in carotid artery stenting only when it is anticipated that the stent cannot cross the lesion, due to the associated risk of higher procedural stroke rates.(Naylor et al., 2018) The role of cerebral protection devices is controversial, however proximal protection devices are not currently recommended in patients with advanced common carotid disease.(Naylor et al., 2018)

This technique does however retain some disadvantages and requires careful patient selection to be of benefit. This technique requires two puncture sites, each with the potential for complications such as pseudoaneurysm, haematoma and dissection. This can be readily minimised by the use of ultrasound-guided access and careful single wall puncture techniques, with carotid access complications relatively infrequent in a recent case series.(Kolluri et al., 2013) If the 0.014″ wire cannot be advanced retrogradely past the CCA origin stenosis, options include using a 0.01″ wire, 0.008″ wire or supporting catheters. However, similar to an antegrade approach with an 0.035″ wire, if a wire cannot be advanced pass the stenosis, the procedure will be unsuccessful. Compared to a surgical approach, this technique does not allow for distal control and embolic risk is not fully controlled. The use of an occlusion balloon to achieve haemostasis temporarily occludes CCA/ICA flow, however this occlusion time is shorter than occlusion clamp time during surgical endarterectomy. This technique requires at least two proceduralists and may increase procedure duration, although for appropriate cases this technique can reduce the time required to cross the stenosis compared to an antegrade femoral approach.

## Conclusion

The described antegrade-retrograde rendezvous technique for CCA stenting provides a viable alternative to traditional retrograde CCA or transfemoral approaches. In patients with symptomatic severe CCA origin disease, this technique may enable safer and easier lesion crossing, stent delivery and deployment.

## Data Availability

Source data can be provided on request.
